# Genetic and Antigenic Analysis of the First A/New Caledonia/20/99-like H1N1 Influenza Isolates Reported in the Americas

**DOI:** 10.3201/eid0804.010311

**Published:** 2002-04

**Authors:** Luke T. Daum, Linda C. Canas, Catherine B. Smith, Alexander Klimov, William Huff, William Barnes, Kenton L. Lohman

**Affiliations:** * Brooks Air Force Base, San Antonio, Texas, USA; † Centers for Disease Control and Prevention, Atlanta, Georgia, USA

**Keywords:** Influenza, H1N1, hemagglutinin, HA, nucleotide sequences

## Abstract

From February through May of 1999, 13 cases of *Influenza A virus* (FLUAV), type H1N1 were reported at a Department of Defense influenza surveillance sentinel site in Lima, Peru. Genetic and antigenic analysis by hemagglutination inhibition and direct nucleotide sequencing of the HA1 region of the hemagglutinin gene were performed on two isolates, A/Peru/1641/99 and A/Peru/1798/99. Both isolates were distinct from the Bayern/7/95-like viruses circulating in the Americas and closely related to a Beijing/262/95-like variant, A/New Caledonia/20/99. With the exception of travel-related cases, the detection of these isolates represents the first appearance of New Caledonia/20/99-like viruses in the Americas. Since the characterization of these Peru isolates, a number of New Caledonia/20/99-like viruses have been reported worldwide. For the 2000/01 and 2001/02 influenza seasons, the World Health Organization (WHO) has recommended the inclusion of A/New Caledonia/20/99 as the H1N1 vaccine component for both the southern and northern hemispheres.

Influenza epidemics occur in most parts of the world and typically arise every 2 to 3 years, causing approximately 20,000 deaths above the yearly mortality baseline (*1*). The incidence of recurring epidemics is primarily attributed to the high frequency of mutational changes in the hemagglutinin (HA) and neuraminidase (NA) major surface glycoproteins. The hemagglutinin protein, responsible for viral uptake into host epithelial cells, is also the antigen targeted by antibodies. The HA1 subunit of the H1N1 hemagglutinin protein consists of the globular head and contains five major antibody binding sites: Sa, Sb, Ca_1_, Ca_2_, and Cb (*2*–*5*). Mutational changes (antigenic drift) at these four sites are thought to be driven by selective antibody pressure, which can produce novel strains capable of evading the immunologic response. Monitoring the antigenic drift of viruses in global populations is essential for optimal selection of component strains for the annually updated trivalent influenza vaccine.

Since 1976, the United States Air Force (USAF) has contributed to influenza surveillance through a collaborative partnership with the Centers for Disease Control and Prevention (CDC) and World Heath Organization (WHO) (*6*,*7*). In 1997, the USAF expanded surveillance to include US Navy and Army installations and became the Department of Defense Global Emerging Infections System (DOD-GEIS) (*6*). The DOD-GEIS influenza surveillance network receives samples from training bases, ports of entry, and many military sentinel sites in the United States, Pacific Rim, and Europe, and throughout Central and South America.

Since 1995, the predominant *Influenza A viruses*, type H1N1 isolated in North and South America were closely related to A/Bayern/7/95, while viruses belonging to another genetic and antigenic lineage of H1N1 viruses, A/Beijing/262/95, have remained geographically restricted to Asia (*8*–*10*). During spring 1999, Beijing/262/95-like variants, containing a characteristic deletion mutation at amino acid 134 of the HA gene, were isolated from 13 persons at a DOD-GEIS influenza surveillance site in Lima, Peru. These antigenically distinct H1N1 viruses were closely related to A/New Caledonia/20/99, a recently characterized variant from the Beijing/262/95 lineage. Before these Peru isolates, no circulation of amino acid-134 deletion mutants in the Americas was documented.

## Materials and Methods

### Sources of Specimens

 Influenza throat swab specimens were collected in accordance with the case criteria outlined in Canas et al. (*6*). The criteria included patients with a fever >37.8°C accompanied by cough or sore throat. Throat swabs were taken within 72 hours of the onset of symptoms, placed in viral transport media (MicroTest, M4) (VTM), and delivered via commercial carrier to the clinical laboratory at the Epidemiological Surveillance Division, Brooks Air Force Base. The two influenza isolates used in this study were randomly selected from 13 positive patient samples isolated from February to April 1999 from a Department of Defense sentinel site in Lima, Peru.

### Viral Detection

 All influenza viruses examined in this study were passaged one time through Primary Rhesus Monkey Kidney (PMK) tissue culture (Viro-Med, Minneapolis, MN; BioWhittaker, Walkersville, MD). A total of 500 μl of VTM was injected onto a shell vial containing a coverslip with PMK tissue culture. Cultures were tested for viral infection using the centrifugation-enhanced shell-vial technique that includes a cell monolayer grown on a coverslip contained in a shell vial. Specimens are inoculated onto the cell monolayer, subjected to a low speed centrifugation for 60 minutes, incubated at 35°C for 48 hours to 72 hours and then screened with monoclonal antibodies (Chemicon, Inc., Temecula, CA) to detect the presence of *Influenza A or B viruses*. Aliquots of all specimens used in this study for the purposes of genetic analysis were stored at -80°C until use.

### Antigenic and Genetic Typing

For antigenic analysis, aliquots of each isolate were sent to CDC for characterization in hemagglutiniation inhibition (HI) tests with postinfection ferret antisera as previously described (*11*).

For genetic typing, approximately 1 mL of VTM was used to infect PMK tissue culture (Viro-Med, Minneapolis, MN; BioWhittaker, Walkersville, MD)and incubated at 37°C for 3 to 5 days. RNA from was extracted from a 300 μl aliquot of tissue culture fluid using the High Pure Viral RNA Kit (Boehringer-Mannheim, Indianapolis, IN). Influenza RNA was amplified into double stranded DNA by reverse transcription and polymerase chain reaction (RT-PCR) using the Titan One Step RT-PCR Kit (Boehringer-Mannheim). An 1,187 bp cDNA fragment consisting of the HA1 coding region of influenza A hemagglutinin was amplified using forward primer F6-(5′-AAGCAGGGGAAAATAAAA-3′, mRNA) and reverse primer R1193-(5′-GTAATCCCGTTAATGGCA-3′, vRNA sense). The RT-PCR reaction was performed by using a Perkin Elmer 2400 thermocycler (Forest City, CA). The PCR product was purified using the QIAquick spin column purification (Qiagen, Valencia, CA). Purified HA1 amplicon was cycle sequenced by using the Big Dye Terminator Cycle Sequencing Kit (PE Biosystems) run on a 377-XL automated DNA sequencer according to manufacturer recommendations (PE Biosystems). Four internal oligonucleotide sequencing primers (Table 1) were used to sequence the entire HA1 amplicon in both the forward and reverse directions. Multiple sequence alignments, protein translation, and phylogenetic analysis were performed by using the software package Lasergene (version 3.18) (DNASTAR, Madison, WI). The nucleotide and amino acid sequences for A/Peru/1621/99 and A/Peru/1798/99 are available from Genbank under the accession numbers AF268313 and AF268312, respectively. Additional nucleotide and amino acid sequences characterized by Brooks Air Force Base and depicted in phylogenetic analysis are available from Genbank under the accession numbers AY029287-AY029292.

**Table 1:** Characterization of Influenza A H1N1 (HA1) Sequencing Primers

Primer/nt position	Sequence	% GC^a^	Tm^b^(°C)
H1F-272	5′-AATCATGGTCCTACATTG-3′	38.9	57
H1F-734	5′-ACTACTACTGGACTCTGC-3′	50	58
H1R-365	5′-TTCCTCATACTCGGCGAA-3′	50	58
H1R-1110	5′-CCATCCATCTATCATTCC-3′	44.4	55

^a^GC= (Guanosine (G) + Cytidine (C))/ (Adenosine (A)+ Thymidine (T)+ Guanosine (G) + Cytidine (C)) x 100  ^b^Tm= 81.5 + 16.6 * log [Na+] + 41 * (#G + #C)/length – 500/length where [Na+]= 0.1M.

## Results

### Antigenic Analysis

Antigenic characterization by HI, using postinfection ferret antisera, was performed with reference antigens representing two globally cocirculating *Influenza A virus*, type H1N1 antigenic/genetic lineages (A/Beijing/262/95, which includes the variant A/New Caledonia/20/99; and A/Bayern/07/95, which includes A/Johannesburg/82/96). Both Peru isolates were antigenically related to the A/Beijing/262/95 rather than to the A/Bayern/07/95 lineage (Table 2). Both Peru isolates were antigenically close to the new variant, A/New Caledonia/20/99, that recently evolved from the A/Beijing/262/95 virus. H1 titers of the Peru isolates were within a two-fold difference from the homologous titer demonstrated by A/New Caledonia/20/99 (160 vs. 320), while they were fourfold lower than the A/Beijing/262/95 homologous titer (160 vs. 640).

**Table 2:** Hemagglutination Inhibition Reactions of Influenza A(H1N1) Viruses^a^ *Reference* Ferret Antisera

Reference Antigens	Bei/262	NC/20	Bay/07	Joh/82
A/Beijing/262/95	**640** ^b^	320	20	20
A/New Caledonia/20/99	160	**320**	10	<10
A/Bayern/07/95	40	10	**1280**	1280
A/Johannesburg/82/96	40	10	1280	**1280**
Test Antigens				
A/Peru/1621/99	160	160	<10	<10
A/Peru/1798/99	160	160	<10	<10

^a^Test viruses are considered antigenically similar to the reference strain if the H1 titer is equal or two times lower than the homologous titer of the reference virus. If the difference in H1 titers is 4-fold or higher, the test viruses are considered to be antigenically different from the reference strain. ^b^Bold and underlined values represent homologous H1 titers.

### Genetic Analysis

The complete nucleotide and deduced amino acid sequences of the HA1 of the two Peru isolates were compared with those of the previous vaccine strain, A/Beijing/262/95, and the current vaccine strain, A/New Caledonia/20/99. Based on nucleotide alignments, A/Peru/1621/99 and A/Peru/1798/99 share identical HA1 sequences and are genetically closer (97.9%) to A/New Caledonia/20/99 (97.9%) than they are to the A/Bayern/07/95 (94.8%) or A/Beijing/262/95 (96.8%) reference strains.

**Figure 1:**
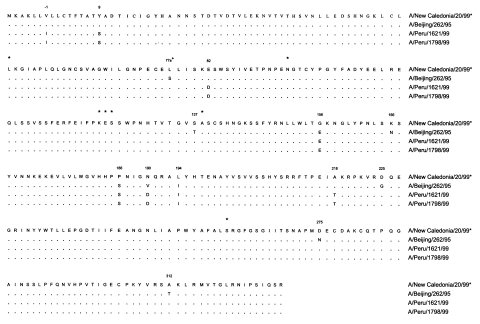
The amino acid sequence of the HA1 region of the hemagglutinin of the Peru isolates and A/Beijing/262/95 compared with the 2000/01 and 2001/02 vaccine strain, A/New Caledonia/20/99. Amino acid numbering corresponds to H3 subtype (*19*), with additional amino acids residues present in the H1 subtype sequence indicated by an asterisk. Residue substitutions are show. A dot (.) indicates amino acid homology to A/New Caledonia/20/99.

A comparison of the amino acid residues of the Peru isolates with A/New Caledonia/20/99 and A/Beijing/262/95 is shown in Figure 1. The Peru isolates contained only 8 amino acid differences compared with A/New Caledonia/20/99 and 11 amino acid differences compared with A/Beijing/262/95. A substitution at residue 156(G156E) shows a neutral to acidic amino acid change similar to those observed in some other Beijing/262-like viruses. Amino acid substitutions at positions 190(N190D) and 194(L194I) are also characteristic of the Beijing/262/95 lineage. Substitutions at position -1 (signal sequence), 9, 82, 186, and 218 represent novel changes not present in A/New Caledonia/20/99 or the majority of characterized Beijing/262-like viruses.

**Figure 2:**
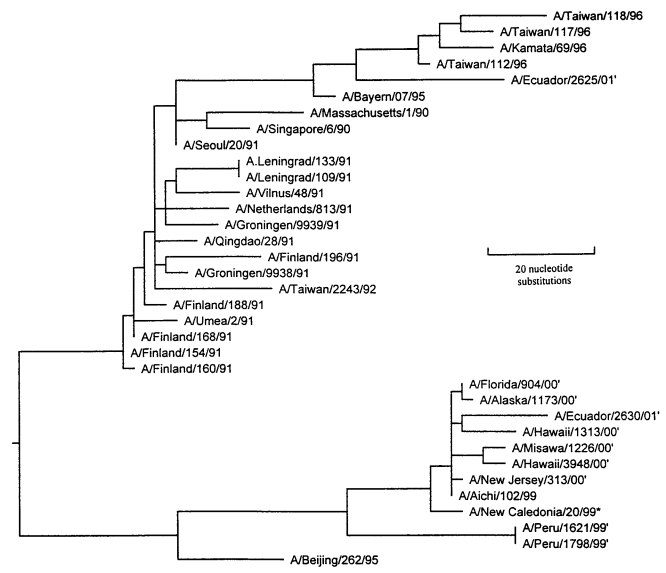
An unrooted phylogenetic analysis of the HA1 gene nucleotide sequence of influenza A H1N1 viruses isolated since 1990. Peru isolates 1621 and 1798 are within the Beijing/262/95 lineage but are more similar to A/New Caledonia/20/99. A number of isolates characterized by Brooks Air Force Base from 1999 to 2001 were also A/NC/20/99-like. The tree was generated by using the Jotun-Hein algorithm (*6*) in MegAlign software (version 3.18). Horizontal lines are proportional to the number of substitutions between branch points. Asterisk (*) denotes 2000/01 vaccine strain; (^1^) denotes isolates characterized by Brooks Air Force Base. Brooks Air Force Base isolates are available from Genbank under accession numbers AF268312, AF268313 and AY029287-AY029292.

A phylogenetic analysis (Figure 2) comparing the HA1 nucleotide sequence of the Peru isolates with H1N1 viruses isolated after 1990 was performed with the software package MegAlign (1993-1998) 0(DNASTAR, Madison, WI), using the Jotun-Hein Method (*12*). Based on the topography, the Peru isolates and other A/New Caledonia/20/99-like viruses isolated by DOD-GIES during the 1999-2000 influenza season appear to have evolved from Beijing/262/95-like viruses into a distinct A/New Caledonia/20/99 sublineage.

## Discussion

A/Beijing/262/95-like viruses first appeared in China during the 1994-95 influenza season and then spread throughout Asia over the next 5 years (*8*–*10*). These viruses have a characteristic deletion mutation in the HA molecule at residue 134. In geographic areas outside Asia, Bayern/7/95-like viruses have predominated during the 1996-97, 1997-98, and 1998-99 seasons (*8*–*10*). In May and June 1999, A/New Caledonia/20/99, an antigenic variant of Beijing/262/95, was first reported in New Caledonia in the Southern Pacific (*13*). Shortly thereafter, A/New Caledonia/20/99-like viruses were detected throughout South Africa and Asia. Isolates A/Peru/1621/99 and A/Peru/1798/99 were collected during spring 1999. These isolates were important because they were the first Beijing/262/95-like viruses in the Americas with the signature HA1 deletion mutation at residue 134. However, the Peru isolates were antigenically distinct from Beijing/262-like viruses and antigenically similar to A/New Caledonia/20/99 (Table 2).

Sequence comparisons further supported the results of the antigenic analysis. Eleven amino acid differences in the HA1 protein were observed between the Peru isolates and A/Beijing/262/95, the vaccine strain for the 1998-99 and 1999-2000 influenza seasons. Four of these 11 substitutions were located in 3 antigenic sites and could potentially alter antibody-binding properties of the viruses. Wilson and Cox proposed that a drift variant of epidemiologic importance usually contains four or more amino acid substitutions located in two or more of the antigenic sites on the HA1 protein (*14*). Substitutions at residues 218(A218T) and 225(G225D) were present in the Ca antigenic site located approximately halfway down the side of the HA1 globular head (*3*,*15*). Two other substitutions, 82(E82D) and 166(N166K), were observed within antigenic sites Cb and Sa, located at the base and tip of the HA1 globular head, respectively (*3*,*15*).

Genetic comparisons between Peru isolates and A/New Caledonia/20/99 show a difference of only eight amino acids (Figure 1). Three of these eight residues, 156(E), 186(S), and 194(I), are present in the A/Beijing/262/95 vaccine strain. The substitution at residue 190(D) is also typical for many Beijing/262/95-like viruses, although not A/Beijing/262/95 itself (*16*). The remaining four substitutions, at residues -1(L), 9(S), 82(D), and 218(T), are unique compared with most recently characterized New Caledonia/20/99 or A/Beijing/262/95-like viruses.

The phylogenetic analysis of *Influenza A viruses* from 1990 to 2001 confirmed the coexistence of the two major H1N1 lineages (Figure 2). The Beijing/262/95-like lineage is easily distinguished from the Bayern/07/95-like viruses by a characteristic deletion at residue 134. With the exception of one Bayern/07/95-like isolate, all the viruses genetically characterized by DOD-GEIS since these Peru isolates were detected contain the residue 134-deletion mutation and appear to be evolving from A/Beijing/262/95 into a distinct sublineage (Figure 2). The A/Peru/1621/99 and A/Peru/1798/99 isolates are depicted as a distinct branch in the phylogeny of New Caledonia-like viruses.

Since these Peru strains were first identified in 1999, DOD-GEIS and CDC have characterized similar New Caledonia/20/99-like viruses isolated from throughout North and South America during the 1999/2000 influenza season. The sudden widespread appearance of these viruses prompted WHO to recommend the change to A/New Caledonia/20/99 as the 2000/01 H1N1 influenza vaccine component (*13*). This recommendation was timely because the 1999/2000 vaccine containing the A/Beijing/262/95 H1N1 component induced a cross-reactive response to A/Bayern/7/95-like viruses but induced lower titers of antibodies to A/New Caledonia/20/99-like strains (*17*). Therefore, a change in the H1N1 component was necessary to ensure that the vaccine would provide effective immunization against emerging A/New Caledonia/20/99-like viruses.

During the 2000/01-influenza season, New Caledonia-like viruses continued to predominate over the H1N1 Bayern-like lineage and were associated with outbreaks in many countries (*13*,*18*). During this season, DOD-GEIS characterized 83 influenza A H1N1 viruses. Of these, 80 (96%) were antigenically related to A/New Caledonia/20/99; three (4%) were Bayern/7/95-like. Our antigenic data are consistent with findings that approximately 84% of H1N1 viruses characterized by CDC during the 2000/2001 influenza season were similar to A/New Caledonia/20/99 (*13*,*18*). The continued persistence of New Caledonia-like viruses in the global population encouraged WHO to maintain A/New Caledonia/20/99 as the H1N1 component for the current 2001/2002 influenza season (*20*).

Studies based on amino acid analyses of viruses from previous years to predict the evolution of future H1N1 epidemic strains would be advantageous as a surveillance tool and could contribute to the vaccine selection process. Using a retrospective approach, Bush et al. determined that specific codons in the HA1 domain of the hemagglutinin gene of human influenza subtype H3 are under positive selection to mutate (*21*,*22*). Certain amino acids in the HA1 of human H1N1 viruses mutate at positively selected codons, giving rise to new viral lineages. The characterization of these Peru variants emphasizes the need to be vigilant in examining sublineage amino acid changes that may be indicators for the emergence of future strains.

The effectiveness of an annually determined trivalent influenza vaccine depends on choosing component strains that offer optimal immunity from the numerous variants in global circulation. In addition, timing is critical because the vaccine strains are selected months before the onset of influenza season to ensure the production of adequate amounts of vaccine by drug manufacturers.

## References

[1] Centers for Disease Control and Prevention. Influenza 2000. Available from: URL: http://www.cdc.gov/ncidod/diseases/flu/fluinfo.htm

[2] Breschkin AM, Ahern J, White DO. Antigenic determinants of influenza virus hemagglutinin. Virology. 1981;113:130–40. 10.1016/0042-6822(81)90142-2 6168097

[3] Caton A, Brownlee GG, Yewdell JW, Gerhard W. The antigenic structure of the influenza virus A/PR/8/34 hemagglutinin (H1 subtype). Cell. 1981;31:417–27. 10.1016/0092-8674(82)90135-0 6186384

[4] Gerhard W, Yewdell J, Frankel ME. Antigenic structure of influenza virus haemagglutinin defined by hybridoma antibodies. Nature. 1981;290:713–7. 10.1038/290713a0 6163993

[5] Caton AJ, Raymond FL, Brownlee GG, Yewdell JW, Gerhard W. Antigenic variation in influenza virus. Biochem Soc Trans. 1983;4:435–41. 10.1042/bst0110435 6194022

[6] Canas LC. The Department of Defense laboratory-based global influenza surveillance system. Mil Med 2000;165:Suppl 2. 10920641

[7] Williams RJ, Cox NJ, Regnery HL, Noah DL, Khan AS, Miller JM, Meeting the challenge of emerging pathogens: the role of the United States Air Force in global influenza surveillance. Mil Med. 1997;162:82–6. 9038023

[8] World Health Organization. Recommended composition of influenza virus vaccines for use in the 1997-1998 season. Wkly Epidemiol Rec. 1997;72:57–64. 9057482

[9] World Health Organization. Recommended composition of influenza virus vaccines for use in the 1998-1999 season. Wkly Epidemiol Rec. 1998;73:56–63. 9523511

[10] World Health Organization. Recommended composition of influenza virus vaccines for use in the 1999-2000 season. Wkly Epidemiol Rec. 1999;74:321–5.10530085

[11] Kendal AP, Pereura MS, Skehel J. Concepts and procedures for laboratory based influenza surveillance. Geneva, Switzerland: World Health Organization, 1982.

[12] Hein JJ. Unified approach to alignment and phylogenies. Methods Enzymol. 1990;183:626–45. 10.1016/0076-6879(90)83041-7 2314296

[13] World Health Organization. Recommended composition of influenza virus vaccines for use in the 2000-2001 season. Wkly Epidemiol Rec. 2000;75:61–8.

[14] Wilson IA, Cox NJ. Structural basis of immune recognition of influenza virus hemagglutinin. Annu Rev Immunol. 1990;8:737–71. 10.1146/annurev.iy.08.040190.003513 2188678

[15] Raymond FL, Caton AJ, Cox NJ, Kendal AP, Brownlee GG. Antigenicity and evolution of influenza J1 haemagglutinin, from 1950-1957 and 1977-1983: Two pathways form one gene. Virology. 1986;148:275–87. 10.1016/0042-6822(86)90325-9 3942036

[16] Xu X, Rocha EP, Regenery HL, Kendal AP, Cox NJ. Genetic and antigenic analysis of influenza a (H1N1) viruses, 1986-1991. Virus Res. 1990;28:37–55. 10.1016/0168-1702(93)90088-5 8493812

[17] Centers for Disease Control and Prevention. Update: influenza activity-United States, 1999-2000. MMWR Morb Mortal Wkly Rep. 2000;283:13.

[18] Centers for Disease Control and Prevention. Influenza activity—United States and Worldwide, April-October. MMWR Morb Mortal Wkly Rep. 2000;49:1006–8. 11097141

[19] Winter G, Fields S, Brownlee GG. Nucleotide sequence of the haemagglutinin gene of influenza virus H1 subtype. Nature. 1981;292:72–5. 10.1038/292072a0 7278968

[20] World Health Organization. Recommended composition of influenza virus vaccines for use in the 2001-2002 season. Wkly Epidemiol Rec. 2001;76:58–61. 11236648

[21] Bush RM, Bender CA, Subbarao K, Cox NJ, Fitch WM. Predicting the evolution of human influenza A. Science. 1999;286:1921–5. 10.1126/science.286.5446.1921 10583948

[22] Fitch WM, Bush RM, Bender CA, Cox NJ. Long term trends in the evolution of H(3) HA1 human influenza type A. Proc Natl Acad Sci U S A. 1997;94:7712–8. 10.1073/pnas.94.15.7712 9223253 PMC33681

